# Supplementary material for: Nonlinear El Niño impacts on the global economy under climate change
Source: Nat Commun. 2023 Sep 21;14:5887. doi: 10.1038/s41467-023-41551-9 (PMC10514271; doi:10.1038/s41467-023-41551-9)
Supplement: Supplementary file 1 — Supplementary Information [file 41467_2023_41551_MOESM1_ESM.pdf]

Supplementary Information for
Nonlinear El Niño impacts on the global economy under climate change

Yi Liu[1,2], Wenju Cai[1,2,3*], Xiaopei Lin[1,4], Ziguang Li[1,4], Ying Zhang[5*]

1. Physical Oceanography Laboratory/Frontiers Science Center for Deep Ocean Multispheres and Earth System/Sanya Oceanographic Institution, Ocean University of China, Qingdao, China
2. CSIRO Environment, Hobart, Tasmania, Australia
3. State Key Laboratory of Loess and Quaternary Geology, Institute of Earth Environment, Chinese Academy of Sciences, Xi'an, China
4. Laoshan Laboratory, Qingdao, China
5. School of Management, Ocean University of China, Qingdao, China

*Correspondence to: Wenju Cai (Wenju.Cai@csiro.au) and Ying Zhang (yzhang@ouc.edu.cn)

This supplementary information includes Figures S1-S10 and Tables S1-S4.

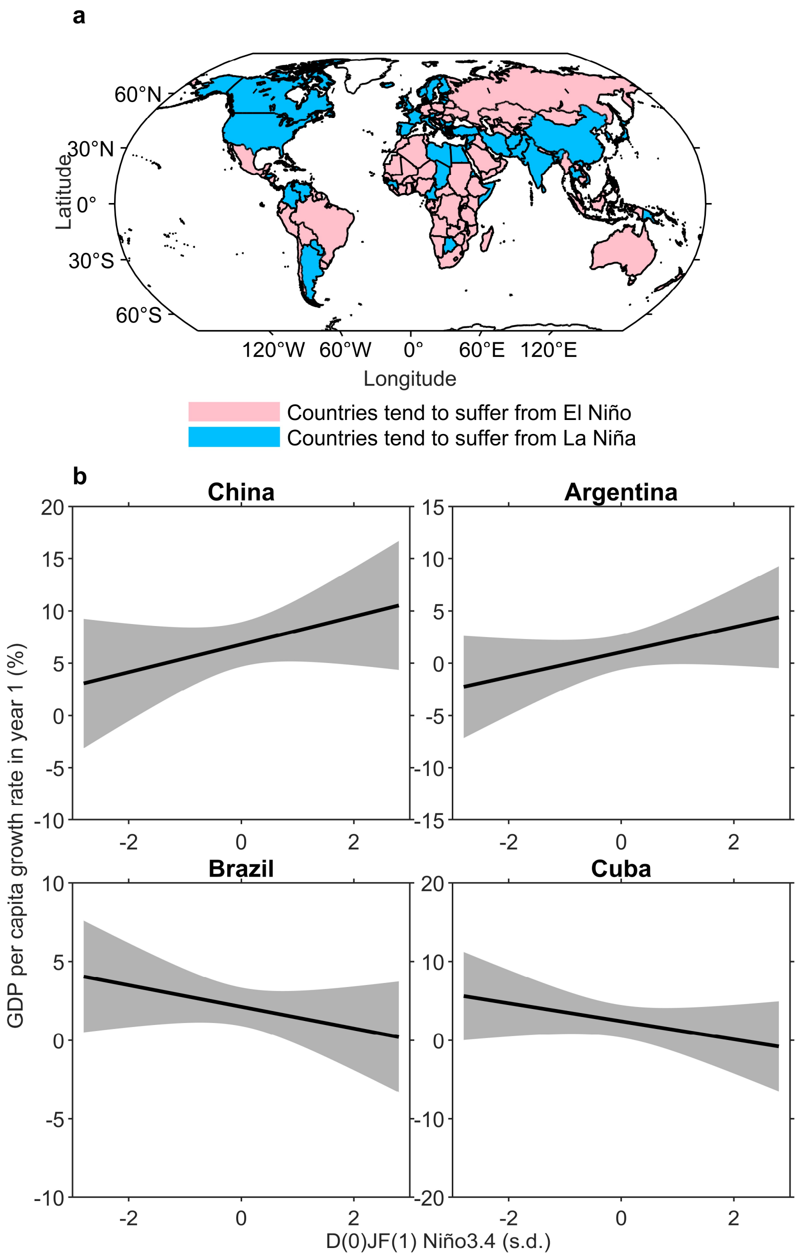

Fig. S1 | Heterogeneous country-level impact from ENSO. a, Classification of countries by whether they tend to suffer economic losses from El Niño (red, negative marginal effect) or La Niña (blue, positive marginal effect). The map is created in the MATLAB computing environment using the M_Map mapping package[1]. **b,** Selected country-level relationships between D(0)JF(1) Niño3.4 index and growth rate in the year after an event over the period of 1960-2019. Black line denotes the fitted linear relationship, with positive slope indicating countries that tend to suffer from La Niña (China and Argentina), and negative slope for countries that tend to suffer from El Niño (Brazil and Cuba); grey area denotes the 95% confidence interval of the fitting.

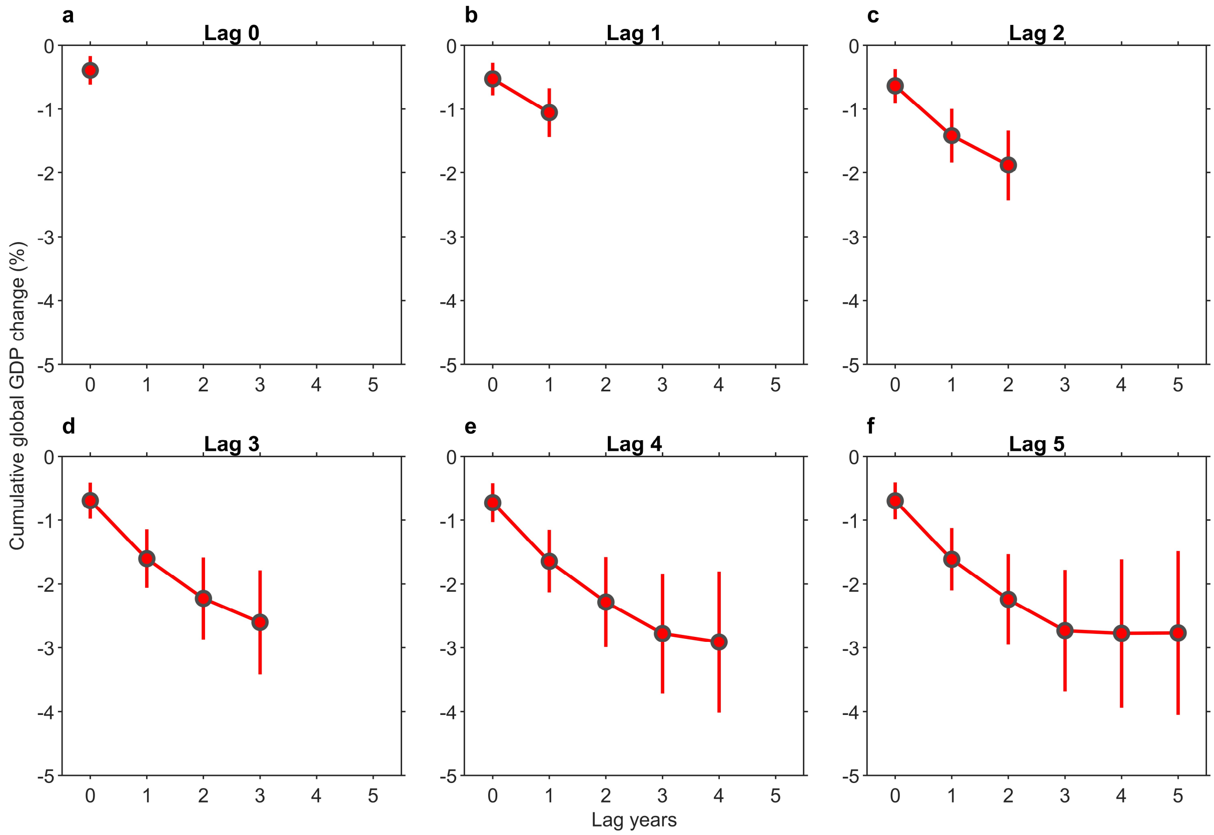

Fig. S2 | Sensitivity of cumulative economic loss to lag years. a-f, Cumulative effect (percentage relative to year -1) of a given El Niño event (D(0)JF(1) Niño3.4 = 1.5 s.d.) on the global GDP growth rate, estimated with **a** 0, **b** 1, **c** 2, **d** 3, **e** 4, and **f** 5 lag years in our econometric model. Error bars indicate the 95% confidence interval at each year based on a Bootstrap method (see 'Statistical significance test' in Methods).

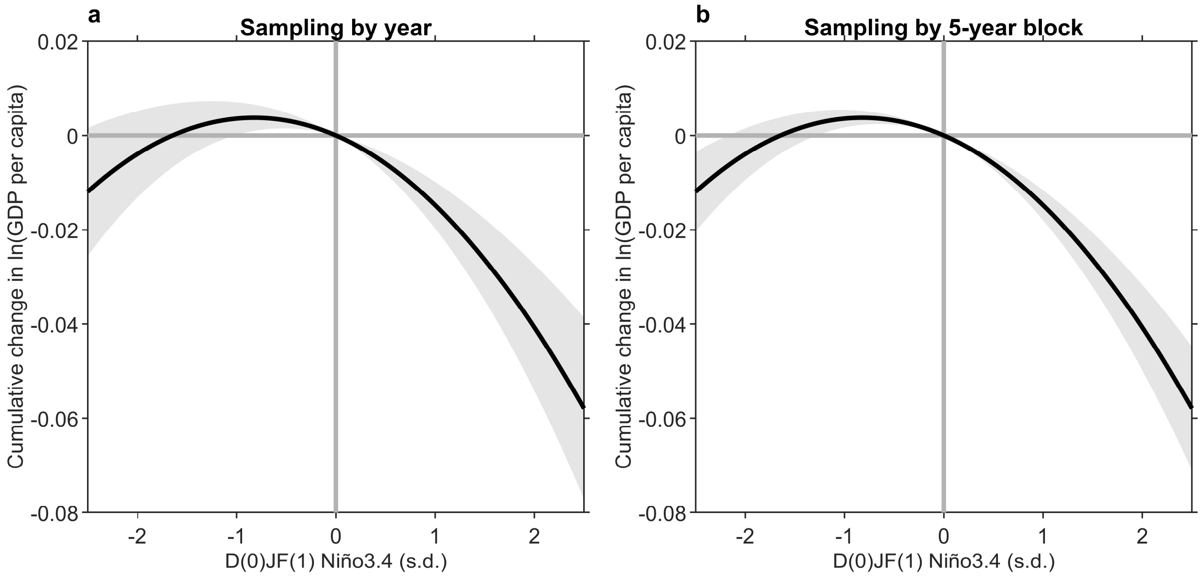

Fig. S3 | Uncertainty of historical response function from alternative bootstrap strategies. a-b, Global nonlinear relationship between D(0)JF(1) Niño3.4 index (normalized) and 3-year cumulative (from year 0 to year 3) change in log GDP per capita for all countries during 1960-2019, with shading indicating the 95% confidence level based on Bootstrap method of **a** sampling by year, and **b** sampling by 5-year block (see 'Statistical significance test' in Methods).

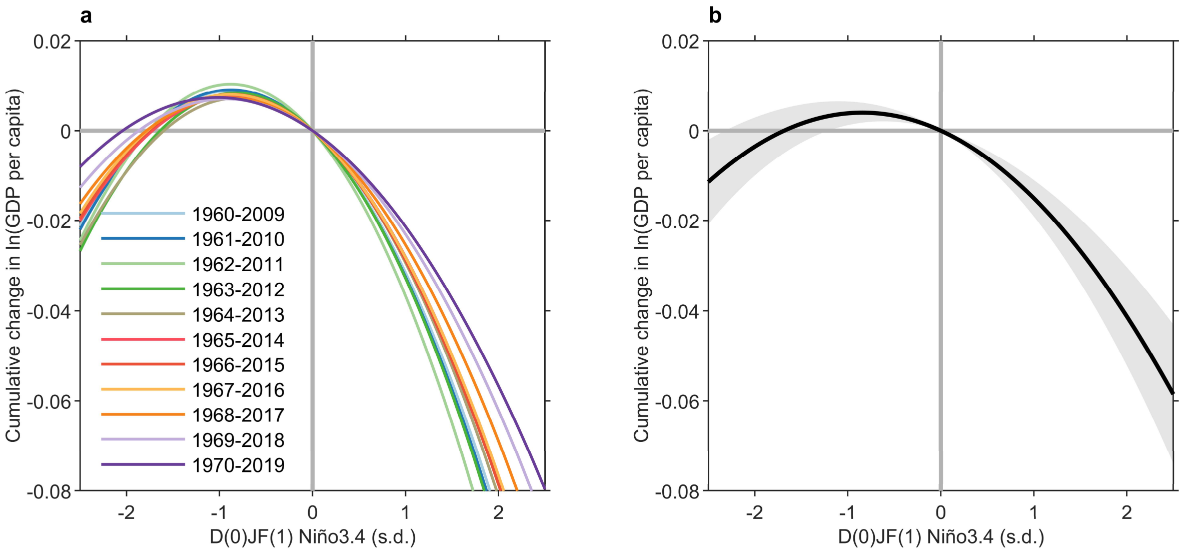

Fig. S4 | Sensitivity to data omitting. Sensitivity test of nonlinear effect of ENSO on global economy for **a** re-estimating by the running-window periods of 1960-2009, 1961-2010, …, and 1970-2019 (colored curves); **b,** re-estimating by randomly dropping 3 individual years from 1960-2019 based on the Bootstrap method. Black curve indicates the original relationship. Shading indicates 95% confidence level from the Bootstrap method (see 'Statistical significance test' in Methods).

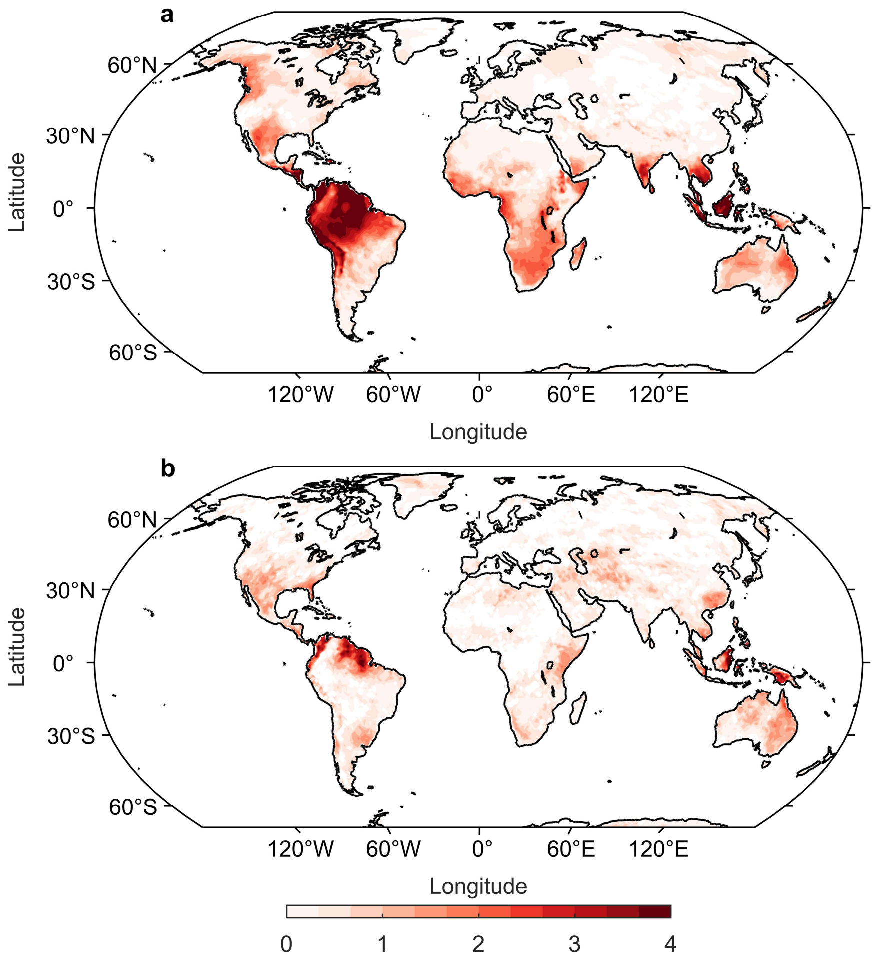

Fig. S5 | Grid-point ENSO teleconnection. Global cumulative grid-point ENSO teleconnection of **a** temperature ($\tau_{x,y}$) and **b** precipitation ($\rho_{x,y}$), calculated as the sum of May(0) to April(1) monthly regression coefficients of normalized surface air temperature and precipitation anomalies onto normalized DJF Niño3.4. The maps are created in the MATLAB computing environment using the M_Map mapping package[1].

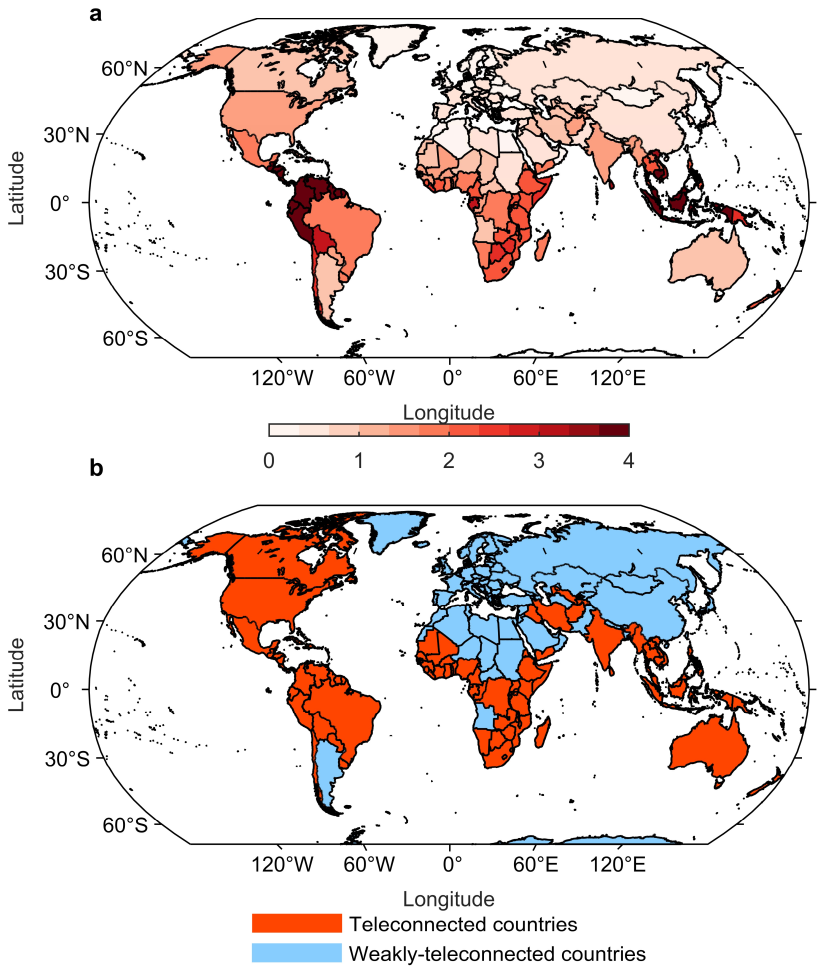

Fig. S6 | Country-specific ENSO teleconnection. a, Country-specific cumulative ENSO teleconnection strength ψ_i, which is calculated as sum of cumulative temperature and precipitation teleconnection strength from May(0) to April(1) that are statistically significant. **b,** Classification of countries into groups of "teleconnected" defined as ψ_i>1 (red), and "weakly-teleconnected" defined as $\psi_i \leq 1$ (blue). The maps are created in the MATLAB computing environment using the M_Map mapping package[1].

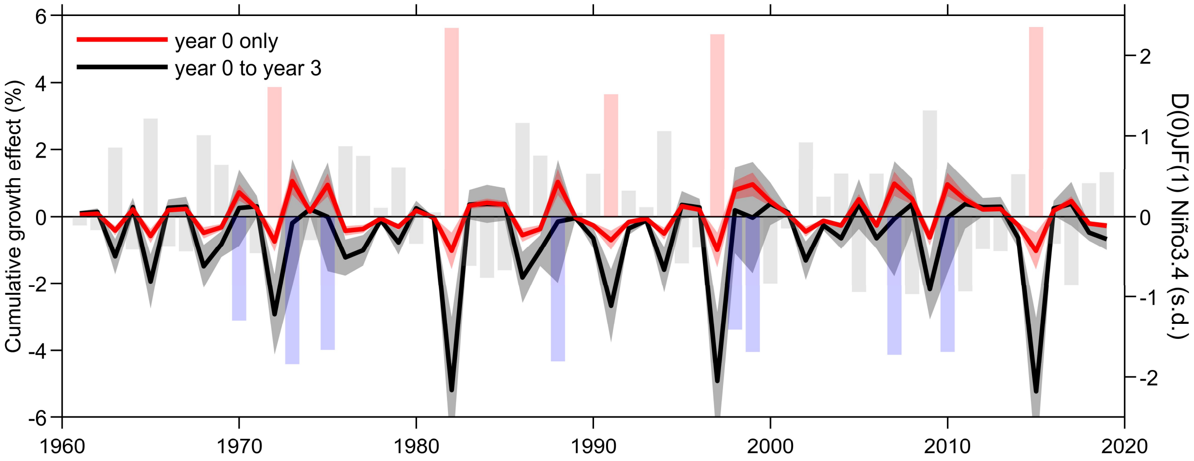

Fig. S7 | Cumulative growth effect of ENSO. Cumulative growth effect of ENSO on global economic production in terms of D(0)JF(1) Niño3.4 at each year. Red and black lines indicate the growth effect only in the ENSO occurrence year and cumulated after subsequent three years, respectively. Shadings show the 95% confidence interval based on a Bootstrap method (see 'Statistical significance test' in Methods). Extreme El Niño (Niño3.4 > 1.5 s.d.) and La Niña (Niño3.4 < -1.25 s.d.) events are marked as red and blue bars, respectively.

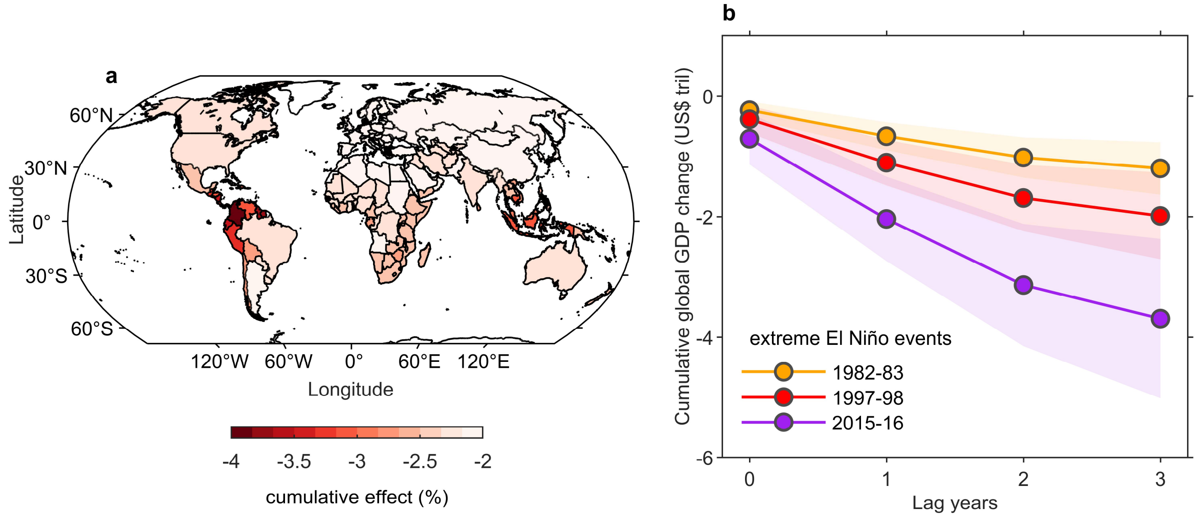

Fig. S8 | Country-specific economic loss from ENSO based on teleconnection. a, Country-specific cumulative effect (percentage relative to year -1) of a given El Niño event (D(0)JF(1) Niño3.4 = 1.5 s.d.) on the country-level GDP growth rate, which is estimated by the econometric model incorporating the interaction of the common ENSO shock with country-specific teleconnection (Eq. 9). The map is created in the MATLAB computing environment using the M_Map mapping package[1]. **b**, Cumulative effect of three major extreme El Niño events in 1982/83 (yellow), 1997/98 (red) and 2015/16 (purple) on the global total GDP under the econometric model with interaction terms included. Shadings indicate the 95% confidence level for each event based on a Bootstrap method (see 'Statistical significance test' in Methods).

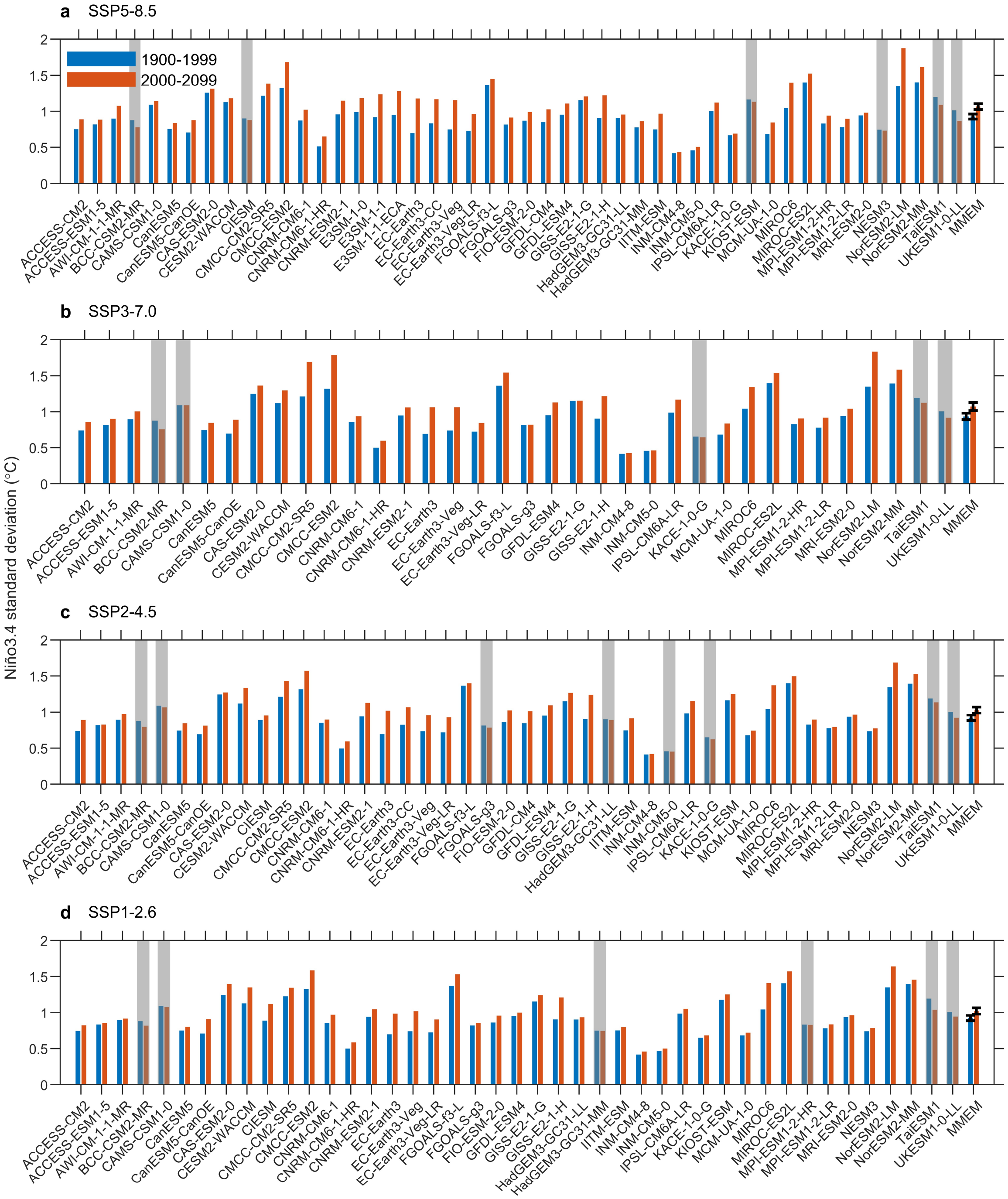

Fig. S9 | Inter-model consensus on increased ENSO SST variability under four IPCC scenarios. a-d, Niño3.4 SST standard deviation (in °C) over the 20th century (1900-1999, blue bars) and 21st century (2000-2099, red bars) for **a** SSP5-8.5, **b** SSP3-7.0, **c** SSP2-4.5, **d** SSP1-2.6 scenarios, with 87.5%, 86.5%, 81.8% and 86.1% models, respectively, simulating an increase in ENSO SST variability. Grey shadings indicate models which do not simulate an increase. Error bars for the multi-model ensemble mean are defined as value of standard deviation of inter-model spread in the 20th and 21st centuries based on a Bootstrap method (see 'Statistical significance test' in Methods).

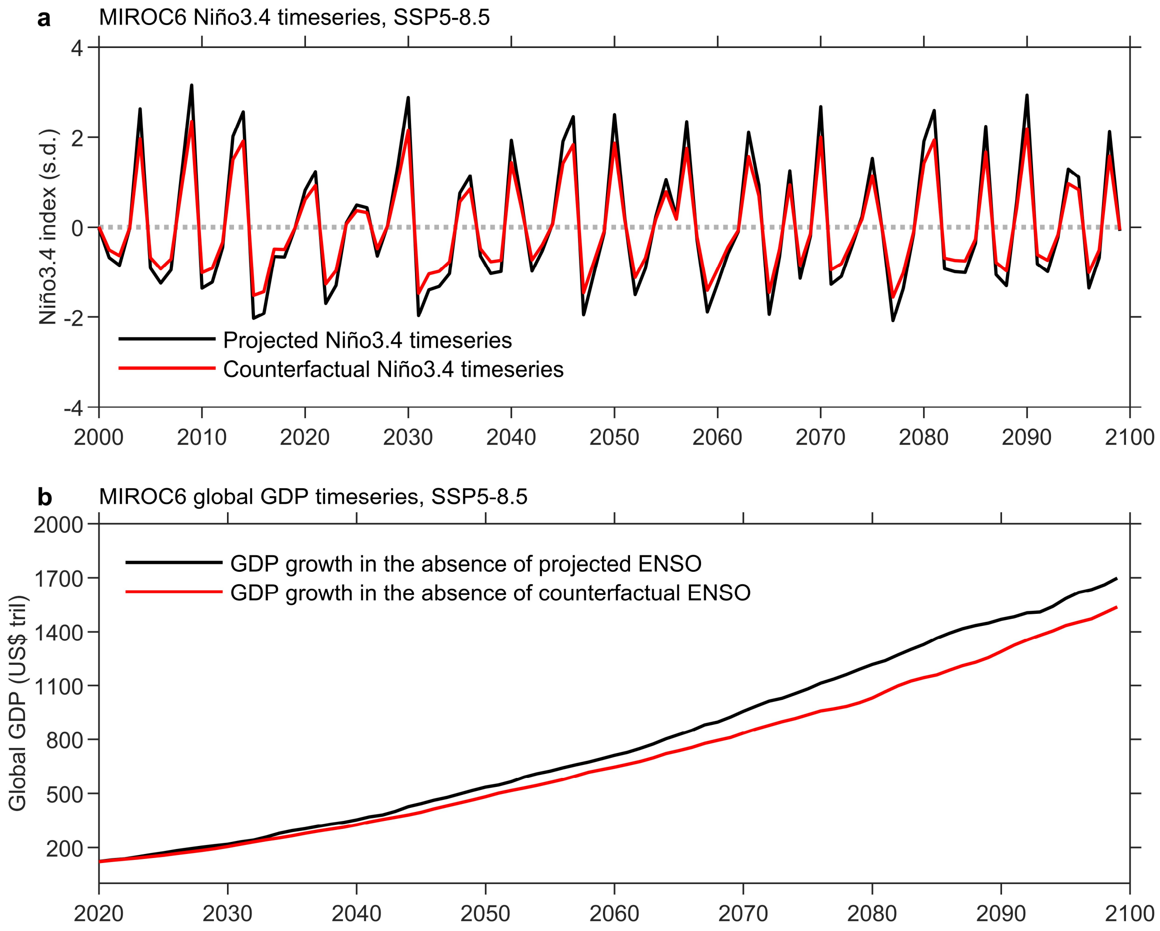

Fig. S10 | Counterfactual ENSO and global GDP timeseries. a, Timeseries of projected (black) and counterfactual (red) Niño3.4 index in the 21st century under the SSP5-8.5 scenario for MIROC6. The counterfactual ENSO is constructed by scaling the amplitude to that of the 20th century to maintain the same sequence of ENSO events (see ‘Counterfactual scenario’ in Methods). **b**, Timeseries of global GDP in the absence of projected ENSO (black) and counterfactual ENSO (red) over the period of 2020-2099 under the SSP5-8.5 scenario for MIROC6. The GDP growth in the absence of ENSO is developed by removing ENSO’s impact.

Table S1 | Regression coefficients of Niño3.4 index, annual mean temperature and precipitation (after removing ENSO's signal) in the econometric model. Niño3.4 index, annual mean temperature and precipitation (after removing ENSO's signal) regression coefficients in various lag years. Superscript *, ** and *** indicate the estimate of coefficient is statistically significant above 90%, 95% and 99% confidence level, respectively.

	**Lag 0**	**Lag 1**	**Lag 2**	**Lag3**	**Lag4**	**Lag5**
$\alpha_{1,0}$	-0.0028***	-0.0037***	-0.0048***	-0.0051***	-0.0047***	-0.0048***
$\alpha_{1,1}$		-0.0011*	-0.0022***	-0.0029***	-0.0030***	-0.0033***
$\alpha_{1,2}$			5.62×10^{-4}	-3.41×10^{-4}	-7.19×10^{-4}	-5.56×10^{-4}
$\alpha_{1,3}$				-8.25×10^{-4}	-0.0014**	-0.0012
$\alpha_{1,4}$					7.17×10^{-4}	9.60×10^{-4}
$\alpha_{1,5}$						-9.21×10^{-4}
$\alpha_{2,0}$	1.46×10^{-4}	9.25×10^{-5}	3.71×10^{-4}	3.08×10^{-4}	-8.99×10^{-5}	-8.00×10^{-5}
$\alpha_{2,1}$		-0.0016***	-0.0021***	-0.0021***	-0.0021***	-0.0019***
$\alpha_{2,2}$			-0.0025***	-0.0026***	-0.0024***	-0.0025***
$\alpha_{2,3}$				-0.0012**	-0.0013**	-0.0014**
$\alpha_{2,4}$					-0.0011*	-8.33×10^{-4}
$\alpha_{2,5}$						6.42×10^{-4}
$\beta_{1,0}$	0.0073***	0.0123***	0.0123***	0.0123***	0.0129***	0.0131***
$\beta_{1,1}$		-0.0093***	-0.0070**	-0.0071**	-0.0077**	-0.0080**
$\beta_{1,2}$			-0.0026	-0.0015	-0.0016	-0.0011
$\beta_{1,3}$				-0.0016	-6.71×10^{-4}	-5.60×10^{-4}
$\beta_{1,4}$					-2.99×10^{-4}	-3.06×10^{-4}
$\beta_{1,5}$						-7.45×10^{-4}
$\beta_{2,0}$	-2.31×10^{-4}***	-3.79×10^{-4}***	-3.71×10^{-4}***	-3.48×10^{-4}***	-3.59×10^{-4}***	-3.63×10^{-4}***
$\beta_{2,1}$		2.78×10^{-4}***	1.88×10^{-4}*	2.13×10^{-4}**	2.42×10^{-4}**	2.49×10^{-4}**
$\beta_{2,2}$			1.14×10^{-4}	1.16×10^{-4}	1.34×10^{-4}	1.16×10^{-4}
$\beta_{2,3}$				-7.23×10^{-5}	-9.83×10^{-5}	-1.08×10^{-4}
$\beta_{2,4}$					-2.90×10^{-5}	-2.92×10^{-5}

$\beta_{2,5}$						1.47×10^{-5}
$\lambda_{1,0}$	-0.0012	-0.0014	-8.10×10^{-4}	-4.84×10^{-4}	-4.01×10^{-4}	-2.68×10^{-4}
$\lambda_{1,1}$		-5.09×10^{-4}	-0.0010	-2.11×10^{-4}	4.49×10^{-4}	4.79×10^{-4}
$\lambda_{1,2}$			-8.09×10^{-4}	1.95×10^{-5}	7.08×10^{-4}	6.52×10^{-4}
$\lambda_{1,3}$				-0.0049	-0.0040	-0.0040
$\lambda_{1,4}$					-0.0063**	-0.0059*
$\lambda_{1,5}$						-0.0035
$\lambda_{2,0}$	-6.47×10^{-6}	7.45×10^{-6}	-1.86×10^{-5}	-4.48×10^{-5}	-5.97×10^{-5}	-6.34×10^{-5}
$\lambda_{2,1}$		4.05×10^{-6}	5.03×10^{-5}	3.84×10^{-5}	-3.13×10^{-5}	-3.05×10^{-5}
$\lambda_{2,2}$			-1.26×10^{-5}	-6.42×10^{-5}	-1.07×10^{-4}	-1.03×10^{-4}
$\lambda_{2,3}$				2.88×10^{-4}	2.27×10^{-4}	2.36×10^{-4}
$\lambda_{2,4}$					3.91×10^{-4}	3.90×10^{-4}
$\lambda_{2,5}$						1.00×10^{-4}
R^2	0.1968	0.1998	0.2025	0.2040	0.2050	0.2056

Table S2 | Regression coefficients of Niño3.4 index in the econometric model for testing the heterogeneity of ENSO effect based on different groups of countries. Same as Table S1, but with regression coefficients of adjustment terms ($\alpha_{3,l}$, $\alpha_{4,l}$) added.

	Teleconnected/Weakly-teleconnected countries	Agriculture-dependent/-independent countries	High-income/Lower-income countries
$\alpha_{1,0}$	-0.0055***	-0.0053***	-0.0053***
$\alpha_{1,1}$	-3.64×10^{-4}	-0.0025***	-0.0032***
$\alpha_{1,2}$	0.0013	4.07×10^{-4}	-0.0014
$\alpha_{1,3}$	5.92×10^{-4}	6.27×10^{-4}	-9.68×10^{-4}
$\alpha_{2,0}$	8.00×10^{-4}	6.00×10^{-4}	8.61×10^{-6}
$\alpha_{2,1}$	-0.0029***	-0.0025***	-0.0024***
$\alpha_{2,2}$	-0.0033***	-0.0029***	-0.0029***
$\alpha_{2,3}$	-4.55×10^{-4}	-8.40×10^{-4}	-0.0017**
$\alpha_{3,0}$	6.05×10^{-4}	6.06×10^{-4}	4.00×10^{-4}
$\alpha_{3,1}$	-0.0046***	-0.0010	7.36×10^{-4}
$\alpha_{3,2}$	-0.0030**	-0.0021	0.0025*
$\alpha_{3,3}$	-0.0026**	-0.0038***	4.02×10^{-4}
$\alpha_{4,0}$	-9.00×10^{-4}	-8.00×10^{-4}	7.28×10^{-4}
$\alpha_{4,1}$	0.0014	0.0011	8.08×10^{-4}
$\alpha_{4,2}$	0.0012	8.44×10^{-4}	7.42×10^{-4}
$\alpha_{4,3}$	-0.0013	-8.74×10^{-4}	0.0013
R^2	0.2063	0.2054	0.2046

Table S3 | Regression coefficients of Niño3.4 index in the econometric model for testing the heterogeneity of ENSO effect based on teleconnection. Same as Table S1, but with regression coefficients of interaction term (γ) added.

	Lag 0	Lag 1	Lag 2	Lag3	Lag4	Lag5
$\alpha_{1,0}$	-0.0032***	-0.0042***	-0.0052***	-0.0055***	-0.0049***	-0.0051***
$\alpha_{1,1}$		-1.84×10^{-4}	-0.0013	-0.0017*	-0.0018*	-0.0022**
$\alpha_{1,2}$			0.0012	4.24×10^{-4}	2.99×10^{-4}	5.57×10^{-4}
$\alpha_{1,3}$				5.25×10^{-4}	-1.12×10^{-4}	3.23×10^{-4}
$\alpha_{1,4}$					0.0021**	0.0025**
$\alpha_{1,5}$						-0.0010
$\alpha_{2,0}$	3.59×10^{-4}	2.52×10^{-4}	5.34×10^{-4}	4.85×10^{-4}	5.07×10^{-5}	2.95×10^{-4}
$\alpha_{2,1}$		-0.0019***	-0.0023***	-0.0024***	-0.0023***	-0.0020***
$\alpha_{2,2}$			-0.0025***	-0.0026***	-0.0024***	-0.0025***
$\alpha_{2,3}$				-0.0012**	-0.0014**	-0.0015**
$\alpha_{2,4}$					-0.0012*	-8.89×10^{-4}
$\alpha_{2,5}$						9.05×10^{-4}
$\gamma_{1,0}$	1.91×10^{-4}	3.13×10^{-4}	2.36×10^{-4}	2.02×10^{-4}	7.62×10^{-5}	1.32×10^{-4}
$\gamma_{1,1}$		-5.19×10^{-4}	-5.06×10^{-4}	-6.85×10^{-4}*	-7.05×10^{-4}*	-6.19×10^{-4}
$\gamma_{1,2}$			-3.54×10^{-4}	-4.46×10^{-4}	-5.88×10^{-4}	-6.36×10^{-4}
$\gamma_{1,3}$				-7.51×10^{-4}**	-7.52×10^{-4}**	-8.75×10^{-4}**
$\gamma_{1,4}$					-7.84×10^{-4}**	-9.01×10^{-4}**
$\gamma_{1,5}$						5.71×10^{-5}
$\gamma_{2,0}$	-2.85×10^{-5}	-2.13×10^{-5}	-2.26×10^{-5}	-2.47×10^{-5}	-2.08×10^{-5}	-3.01×10^{-5}
$\gamma_{2,1}$		3.39×10^{-5}	3.44×10^{-5}	2.90×10^{-5}	2.28×10^{-5}	1.54×10^{-5}
$\gamma_{2,2}$			6.21×10^{-7}	4.62×10^{-6}	-6.46×10^{-6}	-3.16×10^{-6}
$\gamma_{2,3}$				5.08×10^{-6}	7.29×10^{-6}	1.25×10^{-5}
$\gamma_{2,4}$					1.57×10^{-5}	8.37×10^{-6}

$\gamma_{2,5}$						-3.40×10^{-5}
R^2	0.1970	0.2004	0.2031	0.2053	0.2069	0.2076

Table S4 | Information of CMIP6 models used in this study. Names of CMIP6 models, the associated institutions and countries, their ensemble members used in this study (mostly r1i1p1f1, with different ensembles labeled in bold), and unavailable scenarios.

CMIP6 Model	Institute, Country	Ensemble used	Scenario unavailable
ACCESS-CM2	CSIRO, Australia	r1i1p1f1	
ACCESS-ESM1-5	CSIRO, Australia	r1i1p1f1	
AWI-CM-1-1-MR		r1i1p1f1	
BCC-CSM2-MR	BCC, China	r1i1p1f1	
CAMS-CSM1-0	CAMS, China	r1i1p1f1	
CanESM5	CCCMA, Canada	r1i1p1f1	
CanESM5-CanOE	CCCMA, Canada	**r1i1p2f1**	
CAS-ESM2-0	CAS, China	r1i1p1f1	
CESM2-WACCM	NCAR, USA	r1i1p1f1	
CIESM	Tsinghua University, China	r1i1p1f1	SSP3-7.0
CMCC-CM2-SR5	CMCC, Italy	r1i1p1f1	
CMCC-ESM2	CMCC, Italy	r1i1p1f1	
CNRM-CM6-1	CNRM, France	**r1i1p1f2**	
CNRM-CM6-1-HR	CNRM, France	**r1i1p1f2**	
CNRM-ESM2-1	CNRM, France	**r1i1p1f2**	
E3SM-1-0	DOE, USA	r1i1p1f1	SSP1-2.6, SSP2-4.5, SSP3-7.0
E3SM-1-1	DOE, USA	r1i1p1f1	SSP1-2.6, SSP2-4.5, SSP3-7.0
E3SM-1-1-ECA	DOE, USA	r1i1p1f1	SSP1-2.6, SSP2-4.5, SSP3-7.0
EC-Earth3	Europe-wide consortium	r1i1p1f1	
EC-Earth3-CC	Europe-wide consortium	r1i1p1f1	SSP3-7.0
EC-Earth3-Veg	Europe-wide consortium	r1i1p1f1	
EC-Earth3-Veg-LR	Europe-wide consortium	r1i1p1f1	
FGOALS-f3-L	CAS, China	r1i1p1f1	
FGOALS-g3	CAS, China	r1i1p1f1	
FIO-ESM-2-0	FIO, China	r1i1p1f1	SSP3-7.0
GFDL-CM4	NOAA-GFDL, USA	r1i1p1f1	SSP3-7.0
GFDL-ESM4	NOAA-GFDL, USA	r1i1p1f1	
GISS-E2-1-G	NASA/GISS, USA	**r1i1p1f2**	
GISS-E2-1-H	NASA/GISS, USA	**r1i1p1f2**	
HadGEM3-GC31-LL	MOHC, UK	**r1i1p1f3**	SSP3-7.0
HadGEM3-GC31-MM	MOHC, UK	**r1i1p1f3**	SSP2-4.5, SSP3-7.0
IITM-ESM		r1i1p1f1	
INM-CM4-8	INM, Russia	r1i1p1f1	
INM-CM5-0	INM, Russia	r1i1p1f1	
IPSL-CM6A-LR	IPSL, France	r1i1p1f1	

KACE-1-0-G	NIMS-KMA, Korea	r1i1p1f1	
KIOST-ESM	KIOST, Korea	r1i1p1f1	SSP3-7.0
MCM-UA-1-0	UA, USA	**r1i1p1f2**	
MIROC6	JAMSTEC, Japan	r1i1p1f1	
MIROC-ES2L		**r1i1p1f2**	
MPI-ESM1-2-HR	MPI-M, Germany	r1i1p1f1	
MPI-ESM1-2-LR		r1i1p1f1	
MRI-ESM2-0	MRI, Japan	r1i1p1f1	
NESM3	NUIST, China	r1i1p1f1	SSP3-7.0
NorESM2-LM	NCC, Norway	r1i1p1f1	
NorESM2-MM		r1i1p1f1	
TaiESM1		r1i1p1f1	
UKESM1-0-LL	MOHC, UK	**r1i1p1f2**	

References

1. Pawlowicz, R., 2020. "M_Map: A mapping package for MATLAB", version 1.4m, [Computer software], available online at www.eoas.ubc.ca/~rich/map.html.
